# BARRIERS AND FACILITATORS FOR FOREIGN EDUCATED NURSES TO PROVIDE QUALITY LONG-TERM CARE

**DOI:** 10.1093/geroni/igac059.2861

**Published:** 2022-12-20

**Authors:** Roy Thompson, Kirsten Corazzini, Thomas Konrad, Michael Cary, Susan Silva, Eleanor McConnell

**Affiliations:** University of Missouri, Colombia, Missouri, United States; University of New Hampshire, Durham, New Hampshire, United States; University of North Carolina at Chapel Hill, Chapel Hill, North Carolina, United States; Duke University, Durham, North Carolina, United States; Duke University, Durham, North Carolina, United States; Duke University, Durham, North Carolina, United States

## Abstract

Unprecedented registered nursing shortages in long-term care (LTC) threaten the provision of person-centered care for older adults in the United States (US). LTC facilities recruit Foreign Educated Nurses (FENs) to address shortages, which raises concerns about care quality due to cultural, linguistic and communication differences among nurses; yet studies have not thoroughly explored FENs’ perspectives on these issues. The purpose of this study was to advance our understanding of FENs’ professional experiences as they began employment in LTC by exploring factors that inhibit or facilitate their provision of quality care. This qualitative descriptive study used purposive sampling to recruit FENs through professional organizations. Eligible FENs were ≥ 18 years old, worked ≥1 year in LTC, and represented racial and ethnic minority groups from Low and Middle Income Countries. In-depth narrative interviews, ranging from 45–60 minutes, were conducted. Applying content analysis, a priori and inductive coding generated themes. Participants (n=12) interviewed were all married females. Most were 50–59 years old (41.7%), Asian (75.0%), BSN-prepared (58.3%), and reported 31–50 years of nursing experience (50%). Positive facility characteristics, acculturation, effective workplace integration and positive support from colleagues, residents, and their families facilitated the provision of quality care. Conversely, negative facility characteristics, cultural barriers, discrimination and ineffective workplace integration were barriers to providing quality care. FENs highlighted culturally-sensitive strategies such as providing structured mentorship and preceptorship programs that supported them in providing person-centered care. FENs confirmed the need to address racial and anti-immigrant discrimination for achieving more equitable and inclusive workplaces.

